# Developing a Diagnostic Algorithm for Identifying Vestibular Neuronitis in Acute Dizziness: An Overview of Epidemiology, Pathogenesis, and Evidence-Based Guidelines for Diagnostic Approaches

**DOI:** 10.7759/cureus.78126

**Published:** 2025-01-28

**Authors:** Petros V Vlastarakos, Giorgos Sideris, Eleni Vasileiou, Efterpi Michailidou, Nikolaos Papadimitriou, Dimitrios Palantzas, Konstantina Melissourgou, Evangelos Panagoulis, Panagiotis P Gogoulos, Thomas Nikolopoulos

**Affiliations:** 1 2nd ENT Department, Attikon University Hospital, National and Kapodistrian University of Athens, Athens, GRC; 2 Otolaryngology - Head and Neck Surgery, Inselspital, University Hospital of Bern, Bern, CHE

**Keywords:** diagnostic algorithm, dizziness, head-impulse test, vertigo, vestibular neuronitis

## Abstract

Vestibular neuronitis (VN) is a prevalent peripheral vestibular disorder presenting with sudden unilateral vestibular loss, leading to acute vertigo without associated cochlear or neurological symptoms. Diagnosis remains challenging due to symptom overlap with other vestibular and central disorders. This study reviews the epidemiology, pathogenesis, and diagnostic approaches for VN to propose a streamlined, evidence-based diagnostic algorithm. A comprehensive literature review was conducted, analyzing 114 studies, including randomized controlled trials, systematic reviews, and clinical guidelines. Emphasis is placed on the clinical history and bedside examinations, supported by ancillary tests to confirm the diagnosis and differentiate VN from central and other peripheral causes of vertigo. The proposed algorithm aims to enhance diagnostic precision and support clinical decision-making.

## Introduction and background

Dizziness is an unpleasant disturbance in spatial orientation. Among its four primary manifestations - vertigo, lightheadedness, presyncope, and disequilibrium - vertigo, defined as the false perception of self-motion, accounts for approximately 54% of reported cases in primary care [1-2]. Unlike a standalone condition, vertigo serves as a key symptom of various diseases with differing etiologies [3]. These underlying conditions may be vestibular, either peripheral (originating from the inner ear) or central (arising from the brainstem or cerebellum), internistic (such as vascular or medication-induced causes), or even psychological in nature [3-4].

Vertiginous patients seen in primary care are most commonly diagnosed with benign paroxysmal positional vertigo (BPPV), acute vestibular neuronitis, or Meniere’s disease, highlighting a significant public health burden [5].

Vestibular neuronitis (VN), in particular, is characterized by a sudden unilateral loss of vestibular function in otherwise healthy young or middle-aged adults. The condition was first described by Ruttin in 1908 at the Annual Meeting of the Austrian Otological Society as “Neuritis vestibularis” [6]. The term “Vestibular neuritis” was introduced by Nylen in 1924, while the term “Vestibular neuronitis” first appeared in a seminal paper by Dix and Hallpike in 1952 [7-8].

From a nomenclature perspective, "neuronitis" refers to a lesion affecting the vestibular nerve, vestibular nuclei, or second-order neurons, while "neuritis" would more appropriately describe a lesion limited to the peripheral vestibular nerve [9]. The term "acute unilateral vestibulopathy" has also emerged in recent years as an alternative term for VN.

This study aims to review the epidemiology, etiology, pathophysiology, and diagnostic approaches for VN, providing a critical assessment of the related evidence.

## Review

Materials and methods

A comprehensive literature search was conducted in Medline, Scopus, CINAHL, and Google Scholar up to December 2024, focusing on three primary objectives: a) identifying guidelines for the diagnosis of VN, b) understanding the pathogenesis of VN, and c) determining the incidence of VN.

The search employed specific keywords and Boolean operators tailored to each database. The primary search strings included: "vestibular neuronitis" OR "vestibular neuritis" OR "acute unilateral vestibulopathy" AND ("aetiology" OR "pathophysiology" OR "diagnosis"), "vertigo" AND "guidelines", "vestibular neuronitis" AND ("systematic review" OR "randomized controlled trial"). Filters such as "guidelines," "randomized controlled trials," and "systematic reviews" were applied to refine the results. Reference lists of retrieved articles were also manually reviewed to identify additional studies. No restrictions were applied based on language during study selection to ensure inclusivity. However, studies without an abstract or with limited accessibility (e.g., unavailable translations) were excluded.

The levels of evidence regarding the primary research question in studies that investigate the results of a treatment are presented in Table 1.

**Table 1:** Levels of evidence regarding the primary research question in studies that investigate the results of a treatment

Category of evidence	Study design
Level I	• high-quality randomized trial with statistically significant difference, or no statistically significant difference but narrow confidence intervals • systematic review of Level I randomized control trials (and study results were homogenous)
Level II	• lesser quality randomized control trial (e.g. < 80% follow-up, no blinding, or improper randomization) • prospective comparative study • systematic review of Level II studies or Level I studies with inconsistent results
Level III	• case-control study • retrospective comparative study • systematic review of Level III studies
Level IV	• case series
Level V	• expert opinion

A risk of bias assessment was performed for all included studies using established evidence-based guidelines and the strength of recommendation by category of evidence for guideline development is presented in Table 2 [10].

**Table 2:** Strength of recommendation by category of evidence for guideline development

Strength of recommendation	Category of evidence
A	directly based on category I evidence
B	directly based on category II evidence or extrapolated recommendation from category I evidence
C	directly based on category III evidence or extrapolated recommendation from category I or II evidence
D	directly based on category IV evidence or extrapolated recommendation from category I, II or III evidence

All retrieved studies were screened based on their relevance to the research objectives. The selection process aimed to identify primary sources for diagnostic guidelines, pathophysiology, and incidence of VN. Secondary outcomes included a) A critical assessment of potential diagnostic actions for acute dizziness, and b) The development of an algorithm for identifying VN in patients presenting with dizziness [11-14].

The search yielded the following: a) Guidelines: Three VN-specific guidelines addressing nomenclature, testing techniques, and a general overview were included, b) Randomized Controlled Trials (RCTs): Of eight RCTs retrieved, only one met inclusion criteria, as others focused on treatment or rehabilitation, c) Excluded Studies: Forty-two studies were excluded for reasons such as lack of abstracts, focus on chronic outcomes, or failure to meet methodological criteria [15-18].

Despite the comprehensive search strategy, some studies were excluded due to a lack of accessible translations or abstracts. Future efforts could benefit from broader access to multilingual databases and translation tools.

Results and discussion

A total of 114 studies were included. The potential diagnostic approaches for patients with acute dizziness are outlined in Table 3.

**Table 3:** Diagnostic actions for patients with acute dizziness

Statement involving the diagnostic pathway	Category of evidence	Strength of recommendation
History-taking is a key diagnostic action when dealing with a patient with acute vestibular symptomatology; ancillary testing is generally of secondary importance in the acute phase [5]	V	-
A normal head-impulse test in a patient with acute nystagmus may be indicative of a central lesion with a pseudo-labyrinthine presentation [11-12]	II	B
The five signs of a central lesion (see text), can allow us to discriminate between a central and a peripheral lesion with a sensitivity and specificity of more than 95% at the bedside [13]	II	B
MRI is generally more appropriate than CT in cases of vertigo, because of its superiority in visualizing the posterior fossa, where most central nervous system diseases that cause vertigo are found [14]	III	C

Epidemiology

Vertiginous syndromes account for 10.7 consultations per 1,000 person-years in UK primary care, while in the USA, 7 million cases are reported annually, with numbers continuing to rise [19-20]. Approximately one-third of the US population has experienced vertigo by age 65, with a lifetime prevalence of rotatory vertigo estimated at 20-30% [21-22].

VN, however, is less common, with an incidence of approximately 3.5 cases per 100,000 people [23]. It typically affects individuals aged 30-60, peaking between 40 and 50 years [22]. Both males and females are equally affected, with a recent common cold reported in 30% of cases prior to onset [24-25]. VN is primarily sporadic, though epidemics have been documented, particularly during winter and spring [26-27]. A second episode of VN occurs in 1.9% of patients, usually affecting the contralateral ear, with no relapses reported in the same ear [28]. The prognosis for VN is generally favorable; however, 15% of patients may develop benign paroxysmal positional vertigo following recovery [29].

Aetiology

Otological principles indicate that diseases involving the inner ear often result in both hearing loss and vertigo due to the impact on cochlear and vestibular components. Based on this, Dix and Hallpike proposed that the absence of measurable hearing loss in patients with VN suggests that the inner ear itself is not affected. Instead, the lesion is likely localized to the vestibular neurons central to the inner ear [9]. However, as there is no evidence of broader brainstem involvement, the lesion appears confined to the vestibular nerve and its connections [30]. Below, the various hypotheses regarding the etiology of VN are outlined.

a) The infectious hypothesis: The association between VN and preceding or concurrent infectious illnesses was first proposed by Dix and Hallpike, who identified a history of infection in 57% of cases [31]. This link has since been supported by additional studies, particularly regarding non-specific upper respiratory tract infections, influenza, and localized infections such as tonsillitis and dental sepsis [8-9,26,32-40]. Sinusitis poses a particularly high risk, reported in up to 50% of cases, while epidemic occurrences further support an infectious etiology [38]. Serological studies have detected markers of recent infections, including influenza viruses A and B, adenoviruses, para-influenza, herpes simplex, Epstein-Barr, rubella, and cytomegalovirus [41-42]. However, no direct viral isolation from blood, respiratory tract, or cerebrospinal fluid has been achieved, and no association with encephalitic viruses has been confirmed [39,42]. The similarities between VN and Bell’s palsy lend credence to the theory of neurotropic viral reactivation, particularly herpes simplex virus type 1 (HSV-1). Polymerase chain reaction (PCR) studies reveal HSV-1 DNA in two-thirds of vestibular and facial ganglia, and experiments demonstrate HSV-1 reactivation in cultured vestibular ganglion cells [43-46]. Despite these findings, the viral infection theory remains circumstantial, supported primarily by elevated neutrophil-to-lymphocyte and platelet-to-lymphocyte ratios in VN patients [47-50]. Evidence from surgically removed tissues also suggests that HSV-1 reactivation may contribute to VN through inflammation and vestibular paralysis [51]. During the COVID-19 pandemic, potential links between SARS-CoV-2 and VN have been explored, including post-viral inflammation and HSV-1 reactivation [52]. However, a study of hospital admissions for acute peripheral vestibulopathy found no significant correlation between COVID-19 and VN [53]. Further research is needed to clarify these associations and understand the impacts of SARS-CoV-2 on the vestibular system.

b) The vascular hypothesis: The vascular hypothesis suggests that acute loss of function in the peripheral vestibular organ may result from vascular occlusion or thrombotic events. Hemenway and Lindsay identified thrombosis of a large vessel in the internal auditory meatus as a potential cause, though similar findings were not confirmed in a separate temporal bone study [32,54]. Sahin et al. further implicated vascular thrombosis by noting elevated mean platelet volume in VN patients compared to healthy controls [47]. Additionally, Han et al. reported a high prevalence of carotid plaques in VN patients, suggesting that these plaques might trigger bloodstream disorders, leading to inner ear dysfunction [55]. Serological evidence supports an inflammatory mechanism contributing to vascular occlusion. Increased levels of peripheral blood mononuclear cells and CRP were observed in VN patients [56]. Freedman and Loscalzo found higher levels of CD40(+) monocytes and macrophages in VN patients, with the CD40 receptor and its ligand (CD40L) driving pro-inflammatory cytokine production and promoting microvascular occlusion through platelet-monocyte aggregates [57]. Further support comes from significant COX-2 expression in B lymphocytes of VN patients compared to controls [25]. At the macroscopic level, Chung et al. linked unilateral VN to ipsilateral vertebral artery hypoplasia, suggesting that impaired blood supply predisposes the vestibular labyrinth and nerve to dysfunction [58]. Increased arterial stiffness and metabolic syndrome scores have also been associated with VN, though the role of cardiovascular risk factors remains debated [59-61]. A systematic review by Simões et al. highlighted significant gaps in research on vascular etiologies and future vascular risks in acute unilateral peripheral vestibulopathy, emphasizing the need for further cardiovascular studies in this context [62].

c) The immunological hypothesis: The characteristic delay between the onset of a preceding respiratory tract infection and the development of vertigo, coupled with the inability to directly isolate a virus during the acute phase, suggests that VN might result from immune-mediated collateral damage rather than a direct viral attack on the vestibular nerve [30]. Immune-mediated neurotropism is a well-established complication of infectious fevers. Similar to vaccine-induced brachial neuropathy, which can occasionally follow deltoid immunization, vestibular nerve neuropathy could theoretically occur after influenza vaccination [30,63]. Oh et al. investigated the inflammatory and thrombotic mechanisms underlying acute unilateral vestibulopathy by analyzing gene expression profiles in 10 patients and 10 controls. They identified 57 differentially expressed genes (DEGs), with 50 upregulated and significantly linked to neutrophil-mediated immune pathways. A comparison of complete blood counts in 72 patients and controls revealed elevated neutrophil-to-lymphocyte ratios in the patient group. The authors proposed that neutrophil-mediated immune responses might contribute to VN by inducing inflammatory and thrombotic changes in the vestibular organ [64]. A key factor in immune-mediated vestibular diseases is the dysregulation of the immune system, marked by an imbalance between T-helper (CD4) and T-suppressor (CD8) lymphocytes. A study using monoclonal antibodies found elevated CD4/CD8 ratios in approximately half of the patients with inner ear diseases, with an associated human leukocyte antigen (HLA) class II DR-typing relative risk of 5.2 for VN [65]. This suggests that T-cell ratios and HLA immune-genetics may play a role in immune-mediated VN. Notably, the imbalance seems primarily due to a greater reduction in CD8 cells rather than an increase in CD4 cells [25]. Despite these findings, the hypothesis that VN is immune-mediated remains speculative.

Pathophysiology

The vestibular nerve consists of two branches: the superior branch, which supplies the cristae of the superior and lateral semicircular canals, the macula of the utricle, and the antero-superior part of the macula of the saccule; and the inferior branch, which innervates the crista of the posterior semicircular canal and the main portion of the macula of the saccule. VN predominantly affects the superior division of the vestibular nerve.

The pathophysiology of VN is closely tied to its proposed etiology, supported by serological evidence, animal studies, and ex-vivo models. According to the infectious hypothesis, the anatomical proximity of the facial and vestibular nerves within the internal auditory meatus, along with their associated ganglia (meatal and vestibular), creates a potential pathway for viral particle transport. Some neurons in the meatal ganglion are located within the vestibular ganglion, with input coming from the palate via the greater superficial petrosal nerve [66]. This anatomical connection allows viral particles to travel from the palate to the vestibular ganglion during life.

Macro- and micro-anatomical studies, along with PCR analyses of human vestibular ganglia, have demonstrated structural differences between the superior and inferior branches of the vestibular nerve. The superior branch is 2.4 mm longer than the inferior branch, which in 65% of cases runs within two separate bony canals. Additionally, anastomoses between the facial and cochlear nerves are more common in the superior branch, potentially explaining its higher susceptibility to VN [67].

An animal model of vestibular neuronitis (VN) was established by inoculating HSV-1 into the auricle of mice, inducing vestibular symptoms [47]. Similarly, intra-labyrinthine inoculation of various viral strains in animal experiments has reproduced vestibular symptomatology, with viral antigens identified in the vestibular membranous labyrinth and ganglion cells [43-45].

Human temporal bone studies have further supported the viral hypothesis. In patients with documented VN, degenerated ganglion cells were found in the meatal ganglion, along with fascicles of degenerated axons in the vestibular nerve [68]. Initially, the virus localizes in the meatal ganglion, spreading cell-to-cell to the superior vestibular division over time. Clusters of affected ganglion cells in both ganglia result from this progressive viral spread. When the viral load reaches a critical threshold, reactivation occurs, causing the virus to breach the ganglion cell membrane and initiate focal axonal degeneration. Reactive microglia assume a phagocytic role in the degenerative process [69]. Importantly, age-related changes are unlikely to explain this degeneration, as control temporal bones matched for age and sex showed no similar findings [70].

If the intra-axonal virus flow is directed toward the brainstem, as seen with the H125 strain of HSV-1, VN develops clinically, and trans-synaptic transmission to second-order neurons in the vestibular nuclei becomes possible [70]. Direct evidence for a viral etiology was provided by transmission electron microscopy of excised vestibular nerves from an affected patient, which revealed viral particles in the cytoplasm of vestibular ganglion cells. Viral assembly occurs as proteins pass through the nuclear membrane into the cytoplasm, where fully formed viral particles are enclosed in transport vesicles [59,71].

A common intersection between the infectious and vascular hypotheses is the role of vestibular artery hypoplasia, which may increase susceptibility to severe viral infections due to reduced immunity and impaired neuronal repair resulting from hypo-perfusion [72-74]. Additionally, hypo-perfusion compromises oxidative killing by neutrophils, a critical defense mechanism against pathogens, directly linked to tissue oxygenation [75-77].

The labyrinthine artery, derived from the anterior inferior cerebellar artery (AICA), supplies the inner ear via key branches like the common cochlear (CCA) and anterior vestibular (AVA) arteries. Disruption of AVA blood flow can mimic symptoms of vestibular nerve inflammation. Factors such as smoking, central adiposity, and diabetes are linked to inner ear atherosclerosis, with the inner ear's high metabolic demands making it particularly susceptible to ischemia, unlike the richly collateralized retrocochlear acoustic nerve [62].

Vestibular artery hypoplasia exacerbates this vulnerability by serving as a regional hemodynamic hindrance to the ipsilateral labyrinth and nerve. Vertebro-basilar insufficiency and hypoplasia selectively harm the peripheral vestibular labyrinth due to high energy demands and minimal collateral circulation [76]. The Fahraeus-Lindqvist effect, coupled with asymmetrical junction geometry, further impedes blood flow from the hypoplastic artery into the ipsilateral AICA [73-78].

At the microvascular level, VN patients exhibit increased pro-inflammatory activation of peripheral blood mononuclear cells (PBMCs), with higher levels of TNF-α-positive B lymphocytes and monocytes. TNF-α promotes leukocyte activation, endothelial adherence, and inflammatory cytokine production, contributing to vascular changes. Elevated CD40 expression on monocytes/macrophages also induces platelet-monocyte aggregates, exacerbating thrombotic and inflammatory processes [79-80].

Impaired immune regulation in VN, marked by an imbalance between CD4 and CD8 lymphocytes - primarily a reduction in CD8 cells - can lead to reduced plasma control and activation of "forbidden clones." These clones may produce autoantibodies against the vestibular nerve, further implicating immune-mediated mechanisms in VN pathophysiology [25].

Diagnosis

History-taking is a critical diagnostic step for patients presenting with acute vestibular symptoms, with ancillary testing typically being of secondary importance during the acute phase [4]. The diagnosis of VN is based on three clinical criteria: a) Sudden onset of vertigo, b) Absence of cochlear symptoms (e.g., hearing loss, tinnitus), and c) Absence of associated neurological symptoms [81].

Vertigo is the hallmark symptom, often occurring abruptly and without warning. Accompanying symptoms may include the perception of object movement in the visual field (oscillopsia) and nausea [81]. While the onset is abrupt in 73% of cases, a gradual onset with a brief prodromal phase (e.g., feeling “off balance” for one to two days) is reported in 27% of cases [24,30]. Symptoms frequently begin at night, presenting on waking [3]. The severity of vertigo ranges from mild unsteadiness to debilitating imbalance. It is often accompanied by vegetative symptoms, such as nausea (94% of cases) and vomiting (54%), and is severe enough to prompt hospital visits [40]. Vertigo is typically aggravated by head movements but remains constant, distinguishing it from episodic symptoms seen in conditions like BPPV or Meniere's disease [8,79].

The acute phase of VN generally lasts two to three days but can extend up to a week or more. This is followed by a recovery phase with mild, gradually resolving vertigo or unsteadiness, lasting several days or weeks. The illness typically resolves within six weeks but may extend to nine weeks or longer if central compensation is incomplete [24,51,82-83].

Complementary to the vertiginous attack, there is a deafening absence of associated cochlear and neurological symptomatology in cases of VN. Hence, there is no hearing impairment, intense tinnitus, or aural fullness, which could point to other inner ear diseases. In addition, there is no diplopia, sensory disturbances, dysphagia, dysarthria, limb paralysis, which could suggest central brainstem involvement, or history of migraine, which would justify an associated vestibular symptomatology, or even headache, which may have been caused by brainstem ischaemia, or posterior fossa haemorrhage [5,24,79]. In fact, the patient is usually an otherwise healthy young or middle-aged adult [30].

On clinical examination, a fine, horizontally rotating, spontaneous nystagmus is present in most cases [3,24,33,36-38,79, 83-85]. The direction of nystagmus is towards the non-affected side, and is only present during the acute phase of the disease, resolving roughly within a month from the onset of symptomatology [37]. Nystagmus that does not decrease with visual fixation is classified as non-peripheral vestibular nystagmus. Nevertheless, certain types of central spontaneous nystagmus can occasionally be lessened by fixation, suggesting that the existence of fixation suppression does not eliminate the possibility of central lesions [81]. Spontaneous nystagmus often persists long after the disappearance of vertigo and will remain one year after the onset in 50% of cases. Gaze nystagmus will disappear in about 12 days or three months [85]. If the incident is not very severe, gait deviation to the affected side, along with a tendency to fall towards the affected side can be observed in Unterberger and Romberg’s tests, respectively. The otoscopic findings are normal [81,85].

Examination of the vestibulo-ocular reflex (VOR) via the head-impulse test (HIT) shows impaired function, when the patient turns to the direction of the affected ear, with high-velocity video HIT being superior to the low-velocity one [86]. The VOR responses to head rotations provide information on the neurophysiologic interaction of the vestibular systems of both ears via brainstem pathways, in generating bilateral compensatory eye movements; a normal HIT in a patient with acute nystagmus may be indicative of a lacunar cerebral infarction [87]. In addition, as part of the five signs of a central lesion (big five), including the vertical divergence as a component of the ocular tilt reaction, a gaze-evoked nystagmus contralateral to the direction of the spontaneous nystagmus, saccadic smooth pursuit and a central fixation nystagmus, which is typically not suppressed by visual fixation, it can allow us to discriminate between a central and a peripheral lesion with a sensitivity and specificity of more than 95% at bedside [88-89]. Hence, the combination of three bedside oculomotor tests (horizontal head-impulse test, nystagmus, and test of skew - HINTS) could predict within one minute, and with high sensitivity and specificity (100% and 96%), the peripheral, as opposed to a central, origin of an acute vestibular symptomatology [24, 90-91]. It is worth mentioning that contrary to prior knowledge, a slight skew deviation (SD) occurs in some patients with an acute unilateral peripheral vestibular lesion (presumably due to damage of utriculo-ocular afferents), while large skew deviations (> 3.3 deg), point toward a central lesion [91-92].

Nham et al. conducted a prospective observational study involving 539 patients presenting to the ER with vertigo. They concluded that the combined use of three diagnostic tools - a structured clinical assessment, video head impulse test (vHIT), and video-oculography (VOG) - doubled the diagnostic rate in the ER setting [93].

The differential diagnosis of VN includes five key conditions, particularly in elderly patients. Vertebro-basilar insufficiency (VBI) and brainstem infarction or hemorrhage must be excluded first. VBI is relatively common in older adults, especially those with hypertension, and sudden vertigo is often its primary symptom [94]. In 25% of cases, vertigo may be the sole presenting symptom, though other signs of brainstem involvement (e.g., diplopia, dysarthria, or hemiparesis) often develop over time [79,83].

Meniere’s disease can usually be ruled out due to the absence of associated cochlear symptoms in VN [83]. However, a retrospective study revealed that one-third of patients presenting with isolated acute spontaneous vertigo to an emergency department were ultimately diagnosed with definite or possible Meniere’s disease [95-96]. Positional nystagmus testing, such as the supine head-roll (Pagnini-McClure) test, can aid differentiation. Paretic direction-fixed positional nystagmus was observed in 80% of VN patients and 26% of Meniere’s disease patients, while recovery direction-fixed nystagmus was observed in 14% and 31% of patients, respectively, during the subacute phase [97].

Disequilibrium of aging results from degenerative changes in the peripheral labyrinth, leading to unsteadiness with sudden head movements [98]. However, these symptoms are generally milder and more gradual than those of VN. Ototoxic agents can also cause vertigo but are typically excluded through history-taking. Similarly, acoustic neuroma presents with gradual disequilibrium or unsteadiness, often accompanied by cochlear symptoms, distinguishing it from VN [32].

In younger adults, multiple sclerosis (MS) should be considered, as pseudo-VN has been reported in several cases [99]. While MS rarely presents as isolated vertigo, such a presentation can be difficult to differentiate from VN until further signs of central nervous system involvement become apparent [30,79].

Diagnostic testing that provides a comprehensive assessment of vestibular function and helps differentiate VN from other conditions includes the following: a) Caloric Irrigation: Identifies horizontal semicircular canal hypofunction (canal paresis >25%) or an absence of nystagmic responses on the affected side in superior VN. Inferior VN typically shows normal findings, b) Sensory Organization Test (SOT): Measures postural stability under varying conditions, revealing that vestibular dysfunction impacts balance in all SOT scenarios (subjective symptoms often correlate more strongly with postural instability than with canal dysfunction), c) Subjective Visual Vertical (SVV): Shows a deflection greater than 2.5° toward the dysfunctional side, reflecting afferent changes in the graviceptive pathway due to unilateral vestibular loss and d) Ocular Vestibular-Evoked Myogenic Potentials (oVEMPs): Assesses utricular function. In superior VN, contralesional oVEMPs are absent with normal Cervical Vestibular-Evoked Myogenic Potentials (cVEMPs), while inferior VN shows normal oVEMPs and impaired ipsilesional cVEMPs [24,100-104].

cVEMPs assess the vestibulo-spinal reflex associated with saccular function [105]. In cases involving the superior branch of the vestibular nerve, cVEMPs are typically unaffected in terms of amplitude and latency. However, when the inferior branch is involved, cVEMP responses are absent on the affected side and normal on the contralateral side [106]. The cVEMP pathway includes the inferior vestibular nerve, medial vestibulo-spinal tract, and spinal accessory nerve, culminating in contraction of the ipsilateral sternocleidomastoid muscle. A diagnostic battery combining horizontal and posterior canal video head impulse tests (vHIT) and cVEMPs reportedly identifies superior or inferior neuronitis in all acute cases [107]. Additionally, an abnormal vHIT with low gain, large refixation saccades, and asymmetric oVEMP results can differentiate VN from stroke [108].

Distortion-product otoacoustic emissions (DPOAEs) measure cochlear function and normally show suppression during contralateral sound stimulation, mediated by the medial olivocochlear bundle via the inferior vestibular nerve [109-111]. In cases of inferior vestibular branch involvement, suppression of DPOAE amplitude in the affected ear during contralateral sound stimulation is significantly reduced [112].

Emerging diagnostic approaches include artificial intelligence-based systems for analyzing nystagmic parameters and functional MRI (fMRI) [113-114]. fMRI studies reveal decreased resting-state activity in the contralateral intraparietal sulcus near the supramarginal gyrus during the acute phase of VN [114]. In chronic VN, reduced BOLD signal activity in the primary visual cortex (V1) during visuo-vestibular stimulation correlates negatively with the functional status of patients, indicating long-term vestibular dysfunction [115].

Neuroimaging is recommended for vertiginous patients with neurological symptoms, risk factors for cerebrovascular disease, or progressive unilateral hearing loss [116]. A prospective study showed that high-resolution MRI performed ≥6 months after symptom onset in patients with persistent peripheral vestibular deficits revealed vestibular nerve atrophy, particularly in the superior vestibular nerve, in five of 10 patients [117].

MRI using FLAIR sequences with delayed gadolinium enhancement is preferred over CT for vertigo cases due to its superior visualization of the posterior fossa, where most central nervous system pathologies causing vertigo are located [118]. However, MRI may not always be accessible in emergency settings. Carotid/vertebro-basilar ultrasound can be helpful for diagnosing vascular causes of vertigo [118]. Non-contrast CT has low sensitivity, and MRI may miss approximately 20% of early-stroke cases, making neuroimaging alone insufficient to exclude central causes in patients presenting with acute dizziness or vertigo in emergency departments [119].

The diagnosis of VN is guided by the 2022 criteria from the International Classification Committee for Vestibular Disorders of the Bárány Society: a) Acute or subacute onset of sustained spinning or non-spinning vertigo of moderate to severe intensity lasting at least 24 hours, b) Spontaneous peripheral vestibular nystagmus, typically horizontal-torsional, direction-fixed, and enhanced by removal of visual fixation, c) Clear evidence of reduced VOR function opposite the direction of the fast phase of the nystagmus, d) No acute central neurological or audiological symptoms (e.g., hearing loss, tinnitus, or otalgia), e) No acute central neurological signs (e.g., skew deviation, gaze-evoked nystagmus, or other central ocular motor or vestibular signs) and f) Symptoms not better explained by another condition or disorder [81].

**Figure 1:**
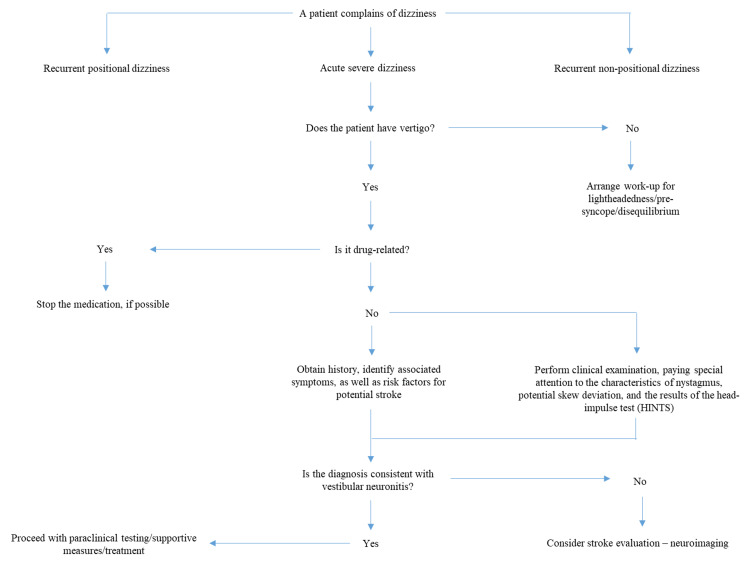
Algorithm for the identification of vestibular neuronitis in patients complaining of dizziness The image has been created by the authors.

According to the results of our study, an algorithm is proposed for the diagnosis of vestibular neuronitis (VN), offering a systematic approach to identifying the condition in patients presenting with acute dizziness (Figure 1).

The algorithm emphasizes a detailed clinical history to confirm the sudden onset of vertigo without associated cochlear or central neurological symptoms. Key bedside tests, including the HIT, spontaneous nystagmus evaluation, and the test of skew, are employed to differentiate VN from central causes of vertigo. Ancillary diagnostics, such as caloric irrigation and VEMPs, provide additional confirmation when necessary, while neuroimaging is reserved for patients with atypical presentations or significant cerebrovascular risk factors. This streamlined approach ensures an accurate and efficient diagnostic process for VN.

## Conclusions

Vestibular neuronitis is a sudden, unilateral vestibular dysfunction that typically resolves with favorable outcomes, albeit with potential long-term vestibular impairment in some cases. Accurate diagnosis relies on clinical history and examination, supported by specific diagnostic tests when necessary. The proposed algorithm simplifies VN identification, emphasizing the importance of distinguishing peripheral vestibular dysfunction from central etiologies. This diagnostic approach seeks to improve patient outcomes and streamline management in clinical settings.
